# Temporal resolution in children: comparing normal hearing, conductive hearing loss and auditory processing disorder

**DOI:** 10.1016/S1808-8694(15)30843-0

**Published:** 2015-10-18

**Authors:** Sheila Andreoli Balen, Letícia Bretzke, Carla Meller Mottecy, Graziela Liebel, Mirian Regina Moresco Boeno, Lys Maria Allenstein Gondim

**Affiliations:** 1 PhD in Neurosciences and Behavior - Universidade de São Paulo, Professor - Speech and Hearing Therapy Program - Universidade do Vale do Itajaí, UNIVALI. Clinical audiologist and speech therapist.; 2 Scholarship holder - artigo 170 do Governo do Estado de Santa Catarina, Student of Speech and Hearing Therapy - Universidade do Vale do Itajaí, UNIVALI.; 3 Speech and Hearing Therapist. Graduate student in Audiology - Universidade do Vale do Itajaí, UNIVALI, Speech and Hearing Therapist - Universidade do Vale do Itajaí, UNIVALI.; 4 Scholarship holder - artigo 170 do Governo do Estado de Santa Catarina, Student of Speech and Hearing Therapy - Universidade do Vale do Itajaí, UNIVALI.; 5 Student of Speech and Hearing Therapy - Universidade do Vale do Itajaí, UNIVALI.; 6 MD. Otorhinolaryngologist - Hearing Department - Universidade do Vale do Itajaí, UNIVALI, Professor - Speech and Hearing Program - Universidade do Vale do Itajaí, UNIVALI. Universidade do Vale do Itajaí.

**Keywords:** hearing, children, hearing loss, auditory perception

## Abstract

Temporal resolution is essential to speech acoustic perception. It may be altered in subjects with auditory disorders, thus impairing the development of spoken and written language. **Aim:** The goal was to compare temporal resolution of children with normal hearing, with those bearing conductive hearing loss and auditory processing disorders. **Materials and methods:** The sample had 31 children, between 7 and 10 years of age, broken down into three groups: G1: 12 subjects with normal hearing; G2: 7 with conductive hearing loss and G3: 12 subjects with auditory processing disorders. This study was clinical and experimental. Selection procedures were: a questionnaire to be answered by the parents/guardians, audiologic and hearing processing evaluation. The study procedure was the test to detect breaks in silence at 50 dB HL above the mean values of 500, 1000 and 2000 Hz in both ears in 500, 1000, 2000 and 4000 Hz. To analyze the data we used the Wilcoxon Test with a 1% significance level. **Results:** We noticed a difference between G1 and G2 and between G1 and G3 in all the frequencies. On the other hand, this difference was not seen between G2 and G3. **Conclusion:** conductive hearing loss and auditory processing disorders can impact break detection thresholds.

## INTRODUCTION

In order for the communication process to be effective among individuals, the speaker must express him/herself properly and make it so that the listener can understand the message.

In listening and decoding what is being said, we can observe the relationship between the peripheral auditory system integrity with the central auditory system. Therefore, in order to have an effective communication, auditory processing effectiveness is paramount^1^.

Auditory processing is associated with what happens when the brain recognizes and interprets sounds^2^. The same thing is defined as the mechanism and process responsible for sound location and lateralization, hearing discrimination, auditory patterns recognition, temporal hearing aspects (temporal resolution, order and sequence), and hearing performance with competitive acoustic signals (including dichotic listening) ^3^.

Auditory processing plays a fundamental role in speech and language development, and a deficit in some of these hearing skills can cause severe speech, reading and writing learning problems to the individual^4^.

Individuals with hearing loss and even those with normal hearing may complain of difficulties understanding speech. This is explained by the auditory processing being incomplete in the periphery of the auditory system; it is not enough to detect sounds, it is necessary to assign some meaning to them. In this context, areas from the central auditory pathway and other systems such as attention, memory, language and thought are involved^5^.

Because of the number of skills being analyzed, it is necessary to have a battery of tests to assess central auditory processing, and each test assesses, mainly, some auditory skill. This test battery must have, at least, dichotic, monaural, low redundancy tests of binaural interaction and temporal processing^6^.

Temporal processing is associated with the perception of the sounds that vary with time, especially in relation to the thresholds of the capacity to detect changes in time^7^. One temporal processing skill is the temporal resolution that can be defined as the capacity the hearing system has to detect the occurrence of two consecutive auditory events and, consequently, avoid that they be detected as a single event^8^.

Numerous researchers say that children’s performance in temporal resolution improves with age^4^, ^5^, ^6^, ^7^, ^8^, ^9^. Children detect longer duration thresholds than adults and will only be similar to the latter at around 10 years of age. Thus, the maturing effects of the central auditory system seem to directly impact their skill to detect small differences in tone duration^12^, ^13^.

Temporal resolution has been investigated in psychoacoustic paradigms since the 70’s; nonetheless, temporal resolution tests were only commercially available in the late 90’s. In Brazil, temporal resolution protocol studies are still very recent and, clinically, are not yet procedures used in the routine evaluation of central auditory processing by all professionals.

Among the temporal resolution evaluation procedures there is the auditory fusion revised test (auditory fusion test-revised - AFT-R) which measures the auditory fusion threshold by the listener’s perception in identifying one stimulus or two, since stimuli duration vary between 0 and 300ms. This threshold is measured for frequencies between 500 and 400Hz15. AFT-R was used in a study with children with reading and writing disorders (RWD) stressing that auditory fusion thresholds are higher in these children when compared to children without RWD^16^. Similar results were found in another study using the AFT-R test in children with cleft palate operated upon and children without a cleft palate, suggesting alterations in the temporal auditory processing^17^. On the other hand, another study showed that there is no evidence of differences in performance in the AFT-R test between genders, age, school variables and the presence of a risk group for language development^18^.

Another, more recent, clinical assessment procedure for temporal resolution is an AFT-R revision called Random Gap detection test - RGDT. Such procedure also uses tones, clicks, and the interval presentation is randomized. The task the individual has to perform in these procedures is to identify whether he/she heard one or two sounds. These are tones or clicks with silence intervals which vary from 0 to 300ms in between the tones^19^.

The original RGDT study was carried out in the United States, with children between 5 and 11 years of age without hearing or school complaints, showed that the mean threshold found in children from five to seven years was 7.3ms (sd = 4.8ms); in 8 year-old children the average threshold was 6.0ms (sd = 2.5ms); in nine year-olds 7.2ms (sd = 5.3ms) and in 10 and 11 year-old children it was 7.8ms (sd = 3.9ms)^20^.

In Brazil, a study with children from private schools in Recife showed a significant difference between the frequencies in which the RGDT was studied; however, there was no difference in relation to gender, age and education^21^. Another study with school-aged children with normal development from Juiz de Fora (MG) found a mean value of 8.7ms (sd = 4.5ms) in silence interval thresholds^22^. Children with speech deviation in tests that assess the temporal auditory processing (tests of frequency patterns and noise interval detection - RGDT) had performances below what was expected, suggesting a difficulty associated with temporal resolution^23^.

The influence of hearing loss caused by a sensorineural problem was broadly investigated in psychoacoustic paradigms of temporal resolution assessment and showed that it does impact temporal resolution, in such a way that individuals with hearing loss have longer duration thresholds when compared to individuals without it^10^. Individuals with conductive hearing loss, when evaluated with high levels of sensation, have interval duration thresholds similar to those from normal hearing individuals^10^, ^11^. Nonetheless, other influences are also reported, such as: age, intensity level of the task and masking noise central frequency.

There are few literature reports about conductive hearing loss influences, as well as which are the temporal resolution test characteristics in children with central auditory processing disorders, considering that the RGDT is still not used in the clinical assessment routine. In this context, the need to study these populations in an attempt to characterize whether its response pattern is clear, in order to guide diagnostic processes which aim at classifying the type of hearing processing disorder present. On the other hand, it helps identify which areas of the central auditory system have dysfunctions and it also helps plan strategies for its rehabilitation^5^.

In the clinical setting, there is also the need to carry out research that show the pros and cons of each temporal resolution protocol, in order to help one chose which would be the most efficient and adequate protocol to use with children. This concern has been the focus of a study carried out in the United States, which aimed at comparing the performance of children with normal development in four temporal resolution tests - auditory fusion test-revised - AFTR; random gap detection test - RGDT; Gaps-in-noise - GIN and the binaural fusion test - BFT. The authors showed that there is a performance difference associated with the task, type of stimulus, presentation and response, and the RGDT and GIN seem to be more advantageous in terms of results and application. Nonetheless, the authors stress that other studies are necessary in order to confirm these findings^24^.

Based on the aforementioned information, this studied aimed at checking the temporal resolution in children with conductive hearing loss (CHL) and with central auditory processing disorders (CAPD).

## MATERIALS AND METHODS

The sample in this paper was made up of 43 children from the 1st to the 4th grades of a Municipal school, as well as children seen in the Audiology Department of the Universidade do Vale do Itajaí, Santa Catarina. These children were invited to participate in this study, and before carrying out the procedures, their parents or guardians were informed about the goals of the study and how it would be done and, after they accepted to participate, they signed an informed consent form, according to the instructions from the Ethics in Research Committee of the Institution, under protocol 145/2006.

This study’s sample had 31 subjects in the age range between 07 and 10 years and 10 months of age, 18 males and 13 females. This population was broken down in three groups: Group 1 (G1): 12 subjects with normal hearing (7:1 to 10:10, mean = 9:05), group 2 (G2): seven subjects with conductive hearing loss (7:0 to 9:0, mean = 7:44) and Group 3 (G3): 12 subjects with auditory processing disorder (central) (7:01 to 10:06, mean = 7:59). Twelve subjects were taken off the study because they did not have the inclusion criteria in the groups or because they did not return in the appointed date to conclude their evaluation.

In order to select the children, the parents and guardians filled out a questionnaire on current and past information about the children, who also underwent audiological and central auditory processing testing. For the audiological assessment, the following procedures were carried out: external ear canal inspection, audiometric screening, tympanometry, and investigation of ipsi and contralateral acoustic reflexes. The children with alterations in the latter were submitted to complete audiological evaluation with: air conduction, bone conduction, speech reception threshold and speech recognition percentage index.

Aiming at diagnosing the central auditory processing disorder, the following procedures were carried out: digit dichotic tests, filtered speech test and Staggered Spondaic Word - SSW. They were all carried out at 50 dB SL above the average of 500, 1000 and 2000Hz^25^.

Starting from these assessments, the group 1 children had the following characteristics:

- no otologic and/or audiologic past problem;
- no school complaints;
- no known neurologic, psychiatric and psychological disorders;
- auditory thresholds within normal values, with type A tympanometric curve and ipsi and contralateral acoustic reflexes, confirmed by audiometric screening through the scanning technique in the frequencies of 500 to 4,000Hz in both ears and auditory thresholds equal to or better than 15 dB;
- uttering of all sounds in Portuguese;
- having read Portuguese as the first and only language;
- no auditory processing disorder, having normal results at the digits dichotic test, alternate dissyllable test and filtered speech test^25^, and
- no hyperactive behavior, lack of attention and/ or impulsiveness.

G2 children, with conductive hearing loss, had the following characteristics:

- Mild and/or moderate hearing loss by the mean values of frequencies 500, 1000 and 2000Hz with types B or C tympanometric curves in at least one of the ears, as well as no contra or ipsilateral acoustic reflex;

G3 children, with central auditory processing disorder, had low results in at least two tests from the central auditory processing battery, as well as complaints and a history of signs and symptoms indicating the disorder^3^. Tonal auditory thresholds and tympanometry were within normal values. The children in this group had schooling complaints and, some children had vocalization disorders.

After the selection procedures, we assessed the temporal resolution - Random gap detection - RGDT. This was carried out through a Compact Disc Player coupled to the Interacoustic AC-33 or AC-40 audiometer. The RGDT had pairs of pure tones in the frequencies of 500, 1,000, 2,000 and 4,000Hz, with intervals between pure tones that varied from 0 to 40ms (RGDT) and from 40 to 300ms (expanded RGDT). The child was instructed to respond with a hand movement if she/he heard one or two tones. The test was carried out at 50 dB SL in binaural tested frequencies. Initially, we carried out the training track 2, if the child detected intervals equal to or below 40ms, we continued in order to measure the frequencies of 500, 1,000, 2,000 and 4,000Hz. If the child did not identify any of the intervals as two tones, the test continued as Expanded RGDT. RGDT result is measured by means of the shortest interval from which the subject started to identify the presence of two stimuli. The result was calculated for each frequency, from 500 to 4,000Hz, and we also calculated the mean result from the four frequencies. The normal value used for comparison was of 6.0 to 7.8ms with 2.5 to 5.3ms of standard deviation in North-American Children^15^.

In analyzing the RGDT, we first checked the number of children who underwent RGDT and the expanded RGDT. Later on we calculated the duration mean and standard deviation at 500, 1,00, 2,000 and 4,000Hz, as well as the duration threshold mean value of all the subjects in each group assessed. For the statistical analysis of the results we applied the Wilcoxon Test, establishing a 0.1 (1%) significance level, because of the small size of the sample in three groups.

## RESULTS

Following, we describe the results attained from all the groups separately and, later on, we present the comparison between both groups.

On table 1, we notice that the silence interval threshold of the sample studied varied from 0 to 30ms depending on the frequency tested, and the thresholds are similar in all the frequencies from G1.

**Table 1:** Descriptive measures of the silence intervals thresholds in G1 (n=12).

Frequency	Mean (ms)	SD (ms)	Median (ms)	Trend (ms)	Minimum-Maximum (ms)
500Hz	11	7,08	10	10	0-20
1,000Hz	8.08	4.72	7.5	10	2-20
2,000Hz	12.83	8.70	15	15	2-25
4,000Hz	12.25	8.68	12.5	15	2-30

Mean	10.94	5.50	11.5	15	0-30

On Table 2 we see the values of silence intervals from G2 - children with conductive hearing loss. In the seven-subject sample, four were submitted to the RGDT and three to the expanded RGDT. We notice little variation in the average among the frequencies tested, however with a lower standard deviation at 1,000Hz. One of the children did not detect 300ms intervals in 4,000Hz and had inconsistent responses.

**Table 2:** Descriptive measures of the silence intervals thresholds in G2 (n= 7).

Frequency	Mean (ms)	SD (ms)	Median (ms)	Trend (ms)	Minimum-maximum (ms)
500Hz	79.29	101.59	30	20	20-300
1,000Hz	63.57	44.97	40	40	25-150
2,000Hz	69.29	75.08	40	10	10-200
4,000Hz*	80.00	87.18	45	40	10-250

Mean	71.19	73.35	36.25	N/D	10-300

* In 4,000Hz the sample had six children; one of them did not recognize the 300ms intervals and gave inconsistent answers.

RGDT results from G3, children with central auditory processing disorders, can be seen on Table 3. This group had a great variability in its intragroup performance, with values varying from 0 to 300ms between minimum and maximum. On Table 3, we notice a lower standard deviation in the frequency of 500Hz and a higher standard deviation at 4,000Hz. In relation to the threshold averages, there was no variation as to the frequency tested.

**Table 3:** Descriptive measures of the silence intervals thresholds in G3 (n=10).

Frequency	Mean (ms)	SD (ms)	Median (ms)	Trend (ms)	Minimum-maximum (ms)
500Hz *	35.63	20.95	50	50	0-250
1,000Hz	41.67	46.30	50	50	0-300
2,000Hz**	41.00	48.09	50	50	0-250
4,000Hz**	51.89	77.86	50	50	0-250

Mean	45.66	42.33	50	50	0-300

* At 500Hz one child did not recognize 300ms intervals.

** At 2,000 and 4,000Hz two children did not recognize 300ms intervals.

**Figure 1:**
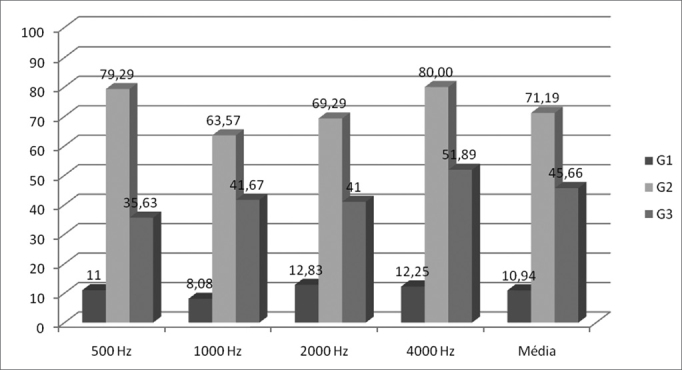
Mean value of the silence intervals thresholds of the groups studied in each frequency in the RGDT test. We noticed a p<0.001 between G1XG2 and G1XG3 and p>0.001 between G2XG3, using the Wilcoxon test.

As we analyze Figure 1, we see the mean values of the silence interval thresholds obtained from all the groups by frequency tested and we notice that the G2 average is higher than that of G3 and, and the latter in relation to G1 in all the frequencies tested. There are statistically significant differences, tested by the use of the Wilcoxon test, between G1 and G2 and between G1 and G3, however we did not see statistically significant differences between G2 and G3.

Thus, we see in this study that the RGDT test is influenced by the conductive hearing loss and the central auditory processing disorder, being a test that differentiates children with normal hearing from those with hearing disorders. On the other hand, the test can not differentiate between the types of hearing disorder: conductive hearing loss and central auditory processing disorder.

## DISCUSSION

The analysis of the results obtained from the RGDT varied according to the group in which the children were classified, there were evidences that the conductive alterations and the central auditory processing disorder generated silence interval detection thresholds above those from the normal group.

We notice that G1 had RGDT mean values similar to that of a Brazilian study^22^, and such average value was greater than the value found for American children^19^. On the other hand, G2 presented higher thresholds than those of children with operated cleft palate and an otologic past^17^. The effect the conductive alteration has on the temporal resolution has been described in the literature as non-existent, because the intensity level is adjusted for the degree of hearing loss^10^, ^11^. Thus, the findings from the present study are different from those from the literature studied. There may be other interfering factors which had not been totally measured at the time, such as audiologic alterations with and without an important otologic past, having seen that the conductive hearing loss is a sensorial privation factor and it can, consequently, generate or prevent the proper development of the central auditory pathways and, thus, also of the temporal resolution skills throughout the child’s development.

Another question regarding temporal resolution skills is the very hypothesis raised by the authors^26^ which states that music learning can indirectly be considered a means of training auditory temporal skills and, consequently, positively interfere in phonologic skills. For that, it is possible to also raise this question in this study, having seen that children with otologic past and/or hearing loss can also have difficulties in learning phonologic skills, as well in temporal processing, thus interfering in the efficient communication of these individuals.

Children from G3 also had interval detection threshold mean values higher than those children from G1 and lower than the G2 ones. Thus, there is evidence about the RGDT validity to diagnose children with central auditory processing disorders. We stress the study on children with reading and writing disorders^27^ in whom we found increased and higher silence interval threshold mean values in comparison to this study. Since the literature shows a close relationship, though not causal, between reading and writing disorders and central auditory processing impairments, it is clear that the RGDT can differentiate normal children from those with CAPD.

Temporal processing, especially temporal resolution, has been described as the basis for specific language disorders^28^ and, it maybe the pathological nature of central auditory processing disorders, showing the need to therapeutically interfere on these skills. Based on scientific evidence, both the American Academy of Audiology^29^ and the American Speech and Hearing Association^3^ suggest the inclusion of temporal resolution procedures in the battery of central auditory processing tests.

When we analyzed threshold mean values by frequency in the RGDT from 500 to 4000Hz in all the groups, we did not see significant differences, showing that both in normal development as well as in the presence of conductive hearing loss and CAPD, the findings are homogeneous among the frequencies^9^. As to the G1, this result is in discordance with the research^10^, ^11^, ^22^ which found longer threshold intervals in the lower frequencies when compared to the high ones during the development.

We stress that the RGDT test showed great performance variability in all the groups studied, and such variability was higher than the one observed in studies with adults^30^. We also see that there were more children with CAPD who needed to undergo expanded RGDT, which corroborates the idea that the RGDT is sensitive enough to detect CAPD. However, qualitative data at the time of test application also hint to the hypothesis regarding the difficulties this group has of understanding instructions given, which is also associated with CAPD difficulties^5^. Thus, one can not totally rule out the alterations presented of understanding nature of the instructions or skill execution, as well as the attention level required by the task, having in mind that the auditory stimuli are fast.

We stress that if the conductive hearing loss is present at the time of the central auditory processing clinical evaluation, the child can present temporal resolution alterations which can not be differentiated from the signs of central auditory processing alterations. Thus, we call the attention of health care professionals and families that the children who are referred for central auditory processing evaluation must present normal bilateral tympanometry at the time of the evaluation.

We need more studies with this protocol as well as a larger study sample in order to further our knowledge about its application and also to characterize it in different populations. It is also necessary to more carefully investigate whether there is or there isn’t influence of the individual’s otologic background besides the momentaneous presence of conductive hearing loss at the time of the temporal resolution development.

## CONCLUSIONS

Based on the results we can conclude that:

• Children with conductive hearing loss have silence interval thresholds, measured by the RGDT test, above those of normal children. Thus, there is an impact of conductive hearing loss in obtaining silence interval thresholds.
• Children with auditory processing disorder (central) show a different performance than normal children, showing that the RGDT test is sensitive to detect these alterations and, therefore, must be included in the battery of tests to assess auditory processing.
• There is no statistically significant difference between silence thresholds of children with central auditory processing disorder and conductive hearing loss. In this case, one must associate the auditory processing assessment (central) at least through tympanometry in order to clear up the diagnosis.
